# Repeatedly Heading a Soccer Ball Does Not Increase Serum Levels of S-100B, a Biochemical Marker of Brain Tissue Damage: an Experimental Study

**DOI:** 10.4137/bmi.s359

**Published:** 2008-02-29

**Authors:** Britt-Marie Stålnacke, Peter Sojka

**Affiliations:** Department of Community Medicine and Rehabilitation (Rehabilitation Medicine), Umeå University, Sweden

**Keywords:** S100 proteins, traumatic brain injury, concussion, sport, soccer

## Abstract

**Objectives:**

The aim of the study was to analyse whether the controlled heading of soccer balls elicits increased serum concentrations of a biochemical marker of brain tissue damage S-100B.

**Methods:**

Nineteen male soccer players were randomly divided into two groups, A and B. Group A headed a soccer ball falling from 18 m five times, while group B served as controls (no heading). Blood samples were taken before and 0.5 h, 2 h and 4 h after the heading for analysis of S-100B.

**Results:**

No statistically significant (p > 0.05) increases in serum concentrations of S-100B were encountered in group A at 0.5 h (0.109 ±0.024 μg/L), 2 h (0.098 ± 0.026 μg/L), and 4 h (0.113 ± 0.035 μg/L) when the blood samples obtained before and after the heading were compared (0.157 ± 0.134 μg/L). No statistically significant difference was found when the serum concentrations of S-100B were compared between groups A and B either before or after heading.

**Conclusions:**

Heading a soccer ball dropped from a height of 18 m five times was not found to cause an increase in serum concentrations of S-100B, indicating that the impact was not sufficient to cause biochemically discernible damage of brain tissue.

## Introduction

Head injuries that affect brain function are very common. Concussions/mild traumatic brain injuries account for the majority (85%–90%) of all head injuries (Jennett and MacMillan, 1981; King, 1997; Thornhill et al. 2000; von Wild and Wenzlaff, 2005) and they have been recognized as a major public concern due to the risk of long lasting symptoms (Rutherford, 1989; King, 1997) and the cumulative effects of multiple head trauma (Gronwall and Wrightson, 1975; Rabadi and Jordan, 2001). Concussions are particularly prevalent in contact sports such as boxing, American football, ice hockey, rugby and soccer (Tegner and Lorentzon, 1996; Delaney et al. 2001; Kelly et al. 2001; Kushner, 2001), where they represent a major medical problem (Kelly et al. 1991; Johnston et al. 2001; Stålnacke et al. 2003). In soccer, concussions are reported to constitute 2%–4.5% of all injuries. The most common cause of concussion in soccer is contact/collision of the player’s head with another object (the ground, the goal-post) or another player’s body (e.g head, elbow, foot), or the ball (Barnes et al. 1998; Kirkendall et al. 2001).

Soccer is unique among sports in that the unprotected head is deliberately used to direct a ball, moving on occasions at a velocity some time of more than 70–80 km/h (Kirkendall et al. 2001) thus possibly subjecting it to peak accelerations of more than 50 g (Naunheim et al. 2000). During recent years, there has been an ongoing debate about whether heading in soccer may cause brain injury. On the one hand several studies have reported cognitive dysfunction (Tysvaer and Lochen, 1991; Matser et al. 2001; Downs and Abwender, 2002) and neurological abnormalities (Tysvaer et al. 1989; Autti et al. 1997) in both retired and active soccer players but on the other hand there are also studies which fail to show any evidence of chronic brain damage in soccer players, in comparison with athletes practicing other sports (Haglund and Eriksson, 1993; Guskiewicz, 2001).

It has been demonstrated that acutely injured brain tissue may “release” substances into the blood where they may be traced. S-100B is probably one of the most frequently investigated substances among such biochemical markers for brain damage. S-100B is a calcium binding protein which is present in high concentrations in the glial cells of the central nervous system. Increased serum concentrations of this marker have been found to reflect the presence and severity of traumatically induced brain tissue damage (Herrmann, 1999; Ingebrigtsen et al. 1999; Elting et al. 2000; Herrmann et al. 2000) In two recent studies, we analysed S-100B from a number of elite female and male soccer players before and after competitive games. We found a significant increase in serum concentration of this marker during game play and the changes in the concentration (post-game minus pre-game values) were found to be significantly correlated to the number of times the players headed the ball (Stålnacke et al. 2004; Stålnacke et al. 2006). We decided, therefore, to investigate in an experimental setting whether controlled heading (a series of 5 headers of a soccer ball) increases the concentration of S-100B in blood, indicating acute damage to brain tissue.

## Methods

Nineteen adult, amateur, male soccer players were recruited from local soccer clubs (age: 22 ± 8 years, length: 179 ± 7 cm, weight: 75 ± 7 kg, number of previous concussions: 1.2 ± 1.6 (range: 0–6); length of time playing soccer: 15 ± 7 years). None of the players performed any physical exercise during the 24 hours preceding the heading session. The players were randomly allocated to one of two groups: A or B. Players in group A (n = 10) were instructed to perform five headers of a ball which was dropped from a height of 18 m, and players in group B (n = 9) served as the control group, as they performed no headers. It was calculated that the ball had reached a velocity of 63.6 km/h upon impact with the head, for the players in group A. This velocity is within the range measured for ordinary kicks (Kirkendall et al. 2001). Standard soccer balls (Puma^R^; n = 20, weight 0.435 ± 0.006 kg) were checked with respect to air pressure just before the tests. The players in group A stood about 5–7 meters from the ball’s expected point of impact with the ground. As the ball was dropped, each player approached the falling ball and performed a header. The players were asked to head the ball as far as possible without jumping and they took it in turns to perform one header at a time. Each player completed the 5 headers in about 15–20 minutes.

Venous blood was sampled before, 0.53 ± 0.06 h, 1.97 ± 0.06 h and 4.02 ± 0.07 h after the heading session. The blood samples were allowed to clot and, after centrifugation and transport, were frozen and stored at −78 °C until analysis. S-100B was determined using a kit for immunoluminometric assay (LIAISON^®^ Sangtec^®^ 100, Sangtec Medical, Bromma, Sweden). The intraasssay coefficient of variance was 6.4% at a mean concentration of 0.11 μg/L, 2.8% at 1.6 μg/L and to 3.6% at 18.4 μg/L (n = 5 measurements at each concentration). The interassay CV was 11% at 0.11 μg/L, 3.7% at 1.6 μg/L, and 3.2% at 18.4 μg/L (n = 5 measurements at each concentration). The detection limit of the test is 0.02 μg/L. All samples were analyzed (as one batch) at the Karolinska Hospital, Department of Clinical Chemistry, Stockholm, Sweden.

The study was approved by the ethics committee of Umeå University.

### Statistical analysis

All data were analysed using the Statistical Package for the Social Sciences (SPSS 14.0.) Non-parametric test were preferred because the samples were small. The Wilcoxon signed ranked test was used for comparison of S-100B concentrations before and after the headers. The Mann-Whitney U-test was used for comparison of concentrations of S-100B between group A (heading group) and group B (controls). The results are expressed as mean value ± standard deviation, unless otherwise indicated. The level of statistical significance was set at p < 0.05.

## Results

Table 1 shows serum concentrations of S-100B in group A (heading group) and in group B (control group) before and at 0.5 h, 2 h and 4 h after the headers.

There were no statistically significant (p > 0.05; Wilcoxon signed ranks test) increases in serum concentrations of S-100B in group A at 0.5 h, 2 h and 4 h respectively after the heading in comparison with the concentrations before the headers. No statistically significant difference (p > 0.05; Mann-Whitney U-test) was found when the S-100B concentrations were compared between groups A and B before the headers or 0.5, 2 h and 4 h after them.

## Discussion

In an attempt to ascertain whether heading a soccer ball causes a rise in the serum levels of the biochemical marker of brain damage, S-100B, we analyzed concentrations before and after a session of controlled heading. No statistically significant changes in the serum levels of S-100B were observed when the concentrations before and after the performance of headers were compared or when the concentrations in subjects who performed headers were compared with control subjects. Consequently, heading a ball falling from a height of 18 m 5 times did not constitute sufficient head trauma to cause any discernable release of S-100B into the blood. Our findings concerning the absence of increases in the serum concentration of S-100B after heading the ball confirm the previous results obtained by Otto et al. (Otto et al. 2000). Our results also support the findings of Zetterberg et al. (Zetterberg et al. 2007) who reported no differences respecting S-100B levels in cerebrospinal fluid between soccer players who had performed headers and control subjects 7–10 days after a heading session.

As our study was conducted using an experimental set-up one can question whether it is representative of ordinary heading in soccer. We calculated that an impact with the head the ball had reached a velocity of 63.6 km/hour for players in group A. This velocity is clearly within the range measured for ordinary kicks (up to 88.5 km/hour) (Kirkendall et al. 2001). Furthermore, in general a player appears to be exposed on average to 5–6 headers during a game (Mehnert et al. 2005). Thus, both the velocity of the ball being headed and the number of headers in the present study represent of what may occur during a soccer game.

It is well known that S-100B reaches peak concentration in blood shortly after the trauma in patients with mild traumatic brain injury and then falls rapidly (Stålnacke et al. 2005; Townend et al. 2006). Similarly, in an animal study, analysis of S-100B in blood after lateral fluid-percussion injury of the brain revealed already significantly increased levels after 30 minutes peaking 2 hours after the trauma (Kleindienst et al. 2005).

Thus, the choice of time points for sampling blood (before and 0.5 hour, 2 hours and 4 hours after the headers) and for the analyses of S-100B appear to be appropriate for discerning any increase.

It is also worth noting that the soccer players in the experimental set-up performed headers without doing any warm-up activities first, while the soccer players in our previous studies were investigated during a competitive game. In those studies we found that game activities (both in men’s and women’s soccer) caused significant increase of serum concentrations of S-100B (Stålnacke et al. 2004; Stålnacke et al. 2006). Moreover, we have also shown increased concentrations of S-100B during the playing of ice hockey and basketball games (Stålnacke et al. 2003). In a study by Kapural et al. (Kapural et al. 2002) iatrogenic disruption of the blood-brain barrier using mannitol, in humans caused increased levels of S-100B in serum thus leading to the suggestion that S-100B was a possible marker of blood-brain-barrier disruption. Since exercise has been shown to cause an increase in blood-brain-barrier permeability in animal studies (Sharma et al. 1991), one possible implication may be that physical exercise during game-associated activities in soccer, ice hockey and basketball may have induced the opening of the blood-brain barrier which might possibly explain the rise of serum concentrations of S-100B (Stålnacke et al. 2003; Stålnacke et al. 2004; Stålnacke et al. 2006).

The absence of any increase in serum concentration of S-100B due to heading in the present study cannot be considered as demonstrating that performing headers does not affect brain tissue. In fact, it is conceivable that more frequent and intense headings (i.e. more than 5 headers and/or heading balls travelling at higher velocities and/or heading involving rotational trauma to the head/brain) are the types of trauma that occur during a soccer game, affect the brain tissue and cause the release of markers reflecting injury to the brain.

Moreover, one can also hypothesise that the exercise associated with playing soccer may influence the permeability of the blood-brain barrier and that a heading-related impact on the brain may then result in the demonstrable release of S-100B into the blood.

In conclusion, heading a soccer ball falling from a height of 18 m 5 times did not seem to influence the S-100B concentration in blood, indicating that the impact on the brain was not sufficient to release S-100B from the brain tissue into the blood.

## Figures and Tables

**Table 1:** The concentrations of S-100B.

Time	Group A heading group (n = 10)	Group B control group (n = 9)	Comparison of S-100B between group A and B
Before the headers	0.157 ± 0.134 μg/L	0.159 ± 0.080 μg/L	p = 0.633
0.5 h after the headers	0.109 ±0.024 μg/L	0.107 ± 0.040 μg/L	p = 0.842
2 h after the headers	0.098 ± 0.026 μg/L	0.102 ± 0.039 μg/L	p = 1.0
4 h after the headers	0.113 ± 0.035 μg/L	0.104 ± 0.035 μg/L	p = 0.780
